# Hydrogenation Properties of the Ti_45_Zr_38−x_Y_x_Ni_17_ (5 ≤ x ≤ 10) and the Ti_45−z_Y_z_Zr_38_Ni_17_ (5 ≤ z ≤ 15) Mechanically Alloyed Materials

**DOI:** 10.3390/ma17204946

**Published:** 2024-10-10

**Authors:** Joanna Czub, Akito Takasaki, Andreas Hoser, Manfred Reehuis, Łukasz Gondek

**Affiliations:** 1 Faculty of Physics and Applied Computer Science, AGH University of Krakow, Mickiewicza 30, 30-059 Krakow, Poland; lgondek@agh.edu.pl; 2 Department of Engineering Science and Mechanics, Shibaura Institute of Technology, Toyosu, Kotoku, Tokyo 135-8548, Japan; takasaki@shibaura-it.ac.jp; 3 Helmholtz-Zentrum Berlin, Hahn-Meitner-Platz 1, 14109 Berlin, Germany; hoser@helmholtz-berlin.de (A.H.); reehuis@helmholtz-berlin.de (M.R.)

**Keywords:** hydrogen-storage materials, amorphous alloys, quasicrystalline alloys, mechanical alloying, neutron diffraction, scanning electron microscopy

## Abstract

The amorphous materials of the Ti_45_Zr_38_Ni_17_ composition synthesized by mechanical alloying are widely recognized for their ability to store hydrogen with gravimetric densities above 2 wt.%. It is also known that those alloys can form a quasicrystalline state after thermal treatment and their structural and hydrogen sorption properties can be altered by doping with various elements. Therefore, in this paper, the results of the studies on the Ti_45_Zr_38_Ni_17_ system with yttrium substituted for titanium and zirconium are presented. We demonstrate that these alloys are able to absorb hydrogen with a concentration of up to 2.7 wt.% while retaining their amorphous structure and they transform into the unique glassy-quasicrystal phase upon annealing. Furthermore, we demonstrate that the in-situ hydrogenation of those new materials is an effortless procedure in which the decomposition of the alloy can be avoided. Moreover, we prove that, in that process, hydrogen does not bind to any specific component of the alloy, which would otherwise cause the formation of simple hydrides or nanoclusters.

## 1. Introduction

Meeting the quick growing demand for green energy is a significant challenge for modern science. The focus is on zero-emission energy sources to prevent environmental pollution, which, therefore, makes hydrogen a promising energy carrier [1,2,3]. Consequently, the production and storage of hydrogen are key issues that need to be resolved [4]. Hence, the pursuit of new hydrogen storage materials continues and, not only that, but tailoring the properties of the already known materials is the subject of research. For instance, nanoengineering and catalysis were reported to improve the hydrogen storage features of the MgH_2_ [5]. Lately, the role of the transition and rare-earth metals in catalysis of the hydrogen storage materials based on Mg/MgH_2_ has been discussed [6].

Lately, extensive research work has been devoted to amorphous hydrogen storage alloys because of their substantial hydrogen storage capability and rapid kinetics of hydrogenation and then dehydrogenation. [7]. Particularly, the amorphous and quasicrystalline alloys with the Ti-Zr and the Ti-Zr-Ni compositions have awoken significant interest because of their possible use in many areas such as coatings [8], shape memory alloys [9], and materials for biotechnology [10]. The properties of those compounds were also intensively studied in past years as targets for neutron generators and definitely for hydrogen storage (electrochemical and gaseous) [11,12,13,14,15,16,17,18,19].

It is known that for the quasicrystalline Ti-Zr-Ni alloys [20], hydrogen saturation can reach 1.6 H/M (the hydrogen-to-metal ratio). On the other hand, challenges with reversibility were outlined for the icosahedral ribbons [21]. It was shown that the Laves phases and the ZrH_2_ appeared as a result of hydrogenation. It was also reported that improved reversibility was reached for the mechanically alloyed and then annealed quasicrystalline Ti_45_Zr_38_Ni_17_ powders. Only minor peaks originating from the Ti_2_Ni phase appeared after hydrogen desorption [16,22]. The reversibility of hydrogen absorption was improved for the Pd-doped Ti-Zr-Ni quasicrystals [23]. Nevertheless, it was shown that the thermal stability of the Ti-Zr-Ni quasicrystals decreases with the addition of Pd [24]. Lee and Kim proved that the equilibrium vapor pressure of hydrogen rose as a result of the substitution of Ti for Pd and V in the quasicrystals of the Ti-Zr-Ni stoichiometry [25]. What is more, the hydrogen storage properties of those quasicrystals were examined through theoretical and numerical analysis [26].

The hydrogen storage properties of the Ti-Zr-Ni and the Zr-Ti-V melt-spun ribbons synthesized by rapid solidification were also studied [27,28,29]. The maximum hydrogen concentration of 3.2 wt.% was obtained for the Ti_53_Zr_27_Ni_20_ and the formation of the hydride resulted in amorphization of the alloy [27]. It was reported by Shahi et al. that an increase in the hydrogen storage capacity of the Ti-Zr-Ni ribbons is affected by the increasing addition of Ti [28]. Then, the reports showed that the Zr-Ti-V ribbons can be effortlessly activated and the corresponding hydrogen absorption kinetics is fast [29]. At the same time, an increase in the Ti content leads to a reduction in the initial rate of hydrogen absorption.

Furthermore, the high entropy alloys within the Ti-Zr-Cr-Mn-Fe-Ni, the Ti-Zr-V-Cr-Ni, and the Zr-Ti-V-Fe systems have been extensively researched recently [30,31,32]. The hydrogen storage properties of such materials are recognized as promising because of the influence of the C14 Laves phase content and the lattice distortion.

Recently, there has been extensive research on the hydrogen storage properties of Ti- and V-based alloys with various chemical compositions, which were synthesized using arc-melting and mechanical alloying techniques, particularly the Ti_0.72_Zr_0.28_Mn_1.6_V_0.4_ [33]; the Ti-V-Mn alloys with Zr, Ni, and Zr_7_Ni_10_ addition [34,35]; the Ti-V-Fe-Zr with various contents of V [36]; and the Ti-V-Mn-Cr with Zr addition [37]. Those studies were aimed at assessing how the alloy composition affects hydrogen sorption.

Equally importantly, we have recently studied the amorphous Ti_45−x_V_x_Zr_38_Ni_17_ (5 ≤ x ≤ 40) material synthesized by mechanical alloying [38]. Moreover, in our previous research work, we reported that hydrogen concentrations of 2.5 wt.% can be reached for the Ti-Zr-Ni alloys with the addition of Mn, Fe, and Co [39,40,41,42]. The main problem that we encountered was the decomposition of the amorphous alloys into simple hydrides during hydrogenation [40]. Lately, that difficulty has been resolved by suitable alterations in the sample activation and the thermodynamics of hydrogenation [39,41,42]. As a consequence, amorphous hydrides and deuterides of the Ti-Zr-Ni and the related alloys can be synthesized with high hydrogen concentrations of 1.4 H/M [41,42].

Doping the Ti-Zr-Ni system with Y is particularly interesting due to the similar electronic configuration of Y, Ti, and Ni. Such substitutions might be the first step toward high entropy alloys (HEAs). However, the reports for the Ti-Zr-Ni alloys with the addition of Y are rare. So far, the microstructure and crystallization behavior of melt-spun Ti_65_Ni_25_Zr_10−x_Y_x_ (x = 0, 2.5, 5, 7.5, and 10) alloys have been researched by Wang et al. [43]. Therefore, in this work, the hydrogenation properties of the Ti_45_Zr_38−x_Y_x_Ni_17_ (x = 5, 10) and the Ti_45−z_Y_z_Zr_38_Ni_17_ (z = 5, 10, 15) alloys are researched for the first time. The aim of this study is to derive the amorphous hydrides/deuterides of the investigated alloys and to follow the transition from the amorphous to the novel glassy-quasicrystalline phase using the in-situ neutron diffraction technique.

## 2. Experimental Process

The amorphous alloys with the composition of Ti_45_Zr_38−x_Y_x_Ni_17_ (x = 5, 10), namely Ti_45_Zr_33_Y_5_Ni_17_ and Ti_45_Zr_28_Y_10_Ni_17_ and Ti_45−z_Y_z_Zr_38_Ni_17_ (z = 5, 10, 15), namely Ti_40_Y_5_Zr_38_Ni_17_, Ti_35_Y_10_Zr_38_Ni_17_, and Ti_30_Y_15_Zr_38_Ni_17_ were synthesized by mechanical alloying using the Frisch Pulverisette 7 planetary mill (FRITSCH GmbH–Milling and Sizing, Idar-Oberstein, Germany). The commercial pure powders of Y with a purity of 99.9% and Ti, Zr, and Ni with a purity of 99.99%, and the proper stoichiometric ratios were put in stainless steel milling bowls of 45 mL with stainless steel balls with diameters of 15 mm. The ball-to-material ratio was 8:1. Milling was performed in the protective Ar atmosphere with a high purity of 99.9999% to avoid spurious oxygen that might reduce the amorphization of the alloys. The experiment lasted 40 h and the ball acceleration was 15 g. Each 30 min. of milling was followed by a 30 min. pause in order to prevent the overheating of the alloy. The amorphicity ratio of the alloys was examined by X-ray powder diffraction (XRD), the results of which will be discussed in the following paragraph. In order to prevent oxidation of the alloys, the samples were stored in airtight containers and the time of their exposure to air was strictly limited.

Also, the microstructure and homogeneity of the synthesized alloys were investigated by scanning electron microscopy (SEM) with microprobe X-ray analysis (EDS). Those experiments were conducted at room temperature with the use of the Jeol 5900 LV microscope (JEOL Ltd., Tokyo, Japan) with the NORAN System SIX microprobe (Thermo Electron Corporation, Waltham, MA, USA).

Hydrogenation of the alloys was conducted with the use of the Sieverts-type PCT-PRO Setaram automated sorption analyzer (SETARAM, Cranbury, NJ, USA). The sample of known mass was placed in an Inconel reactor that was connected to the apparatus. Then, the reactor was flushed several times with high-purity He (99.9999%) and each flushing was followed by the evacuation of the gas. The reactor volume was estimated by the expansion of He from the previously calibrated volumes of the apparatus. Additionally, it was established that the initial activation did not affect the sorption properties of the alloys and, therefore, the samples were not activated prior to the experiment. In the next step, the sample was exposed to H_2_ at 40 bar and 30 °C. Then, the reactor was heated to 200 °C with simultaneous measurement of the pressure for the precise determination of the maximum hydrogen load. Typically, the pressure drop related to H_2_ uptake was observed as above 100 °C. After subsequent annealing at 200 °C for 6 h, the reactor was cooled to 30 °C and the amount of absorbed hydrogen was evaluated. Then, the pressure was decreased to an atmospheric value in order to observe possible desorption at ambient conditions. It was established that desorption was negligibly low, which was later confirmed by the results of the XRD experiments after 2 weeks. The obtained maximum hydrogen concentration was 2.7 wt.% for the Ti_45_Zr_33_Y_5_Ni_17_ and 1.9 wt.% for the Ti_45_Zr_28_Y_10_Ni_17_. The kinetic measurements were performed at a constant temperature equal to 120 °C. The kinetics were tracked with H_2_ applied to the sample at 40 bar after temperature stabilization.

The samples earmarked for the neutron diffraction (ND) experiments were prepared in a similar way; however, deuterium was used instead of hydrogen. The reason for this was that hydrogen exhibits a very high incoherent scattering cross section for neutrons. Preparation of the Ti_45_Zr_38−x_Y_x_Ni_17_ deuterides was performed less than 5 days prior to the ND experiments. The deuterides of the Ti_45−z_Y_z_Zr_38_Ni_17_ were prepared in situ during the experiments. The ND experiments were conducted at the E6 focusing powder diffractometer and the E2 diffractometer (both Helmholtz Zentrum Berlin für Materialen und Energien, SF1-BENSC, Germany) using the incident neutron wavelength of 2.45 Å. The diffraction measurements for the Ti_45_Zr_38−x_Y_x_Ni_17_ alloys and their deuterides and also for the Ti_45−z_Y_z_Zr_38_Ni_17_ deuterides were conducted up to 850 °C with the use of the high-temperature HTF1 furnace (AS Scientific Products, Abingdon, England). Each sample was placed in a quartz tube in an inert high-purity Ar atmosphere. The background data from the furnace and quartz tube were assessed across the entire temperature range, allowing for the extraction of the sample signal from other contributions. For the in-situ synthesis of the Ti_45−z_Y_z_Zr_38_Ni_17_ deuterides, the amorphous powders were enclosed in an Inconel container that was connected to the Sieverts apparatus. The initial pressure of deuterium for that part of the ND experiments was set to 50 bar at 120 °C. Simultaneously, the neutron diffraction patterns were collected with a constant time interval.

## 3. Results

### 3.1. The Microstructure and the Composition

**Figure 1:**
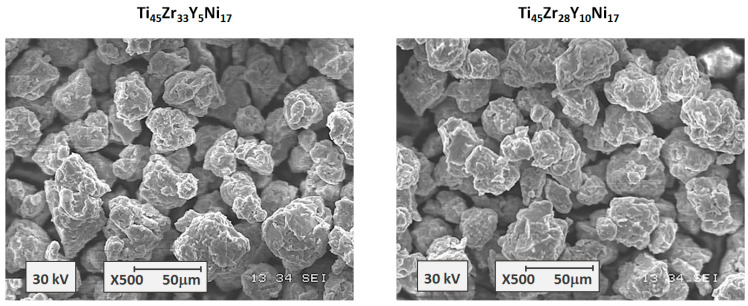
The scanning electron microscopy images for the Ti_45_Zr_38−x_Y_x_Ni_17_ amorphous alloys.

Figure 1 shows the microstructures of the Ti_45_Zr_38−x_Y_x_Ni_17_ alloys with the x = 5 and x = 10, respectively, as seen by the SEM. It can be noticed that the synthesis led to the formation of powders that consist of agglomerates with similar dimensions and diameters of micrometers. Also, agglomerates are built with smaller submicron particles. The microstructures of both specimens are homogenous without large agglomerates.

**Figure 2:**
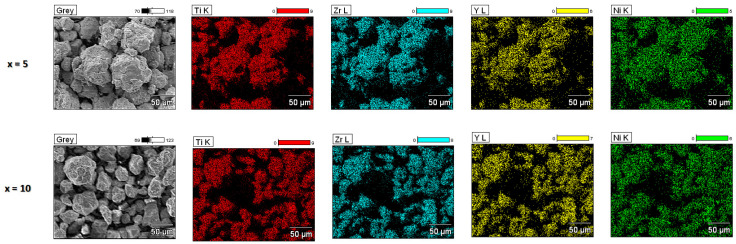
The maps of the elemental distribution for the Ti_45_Zr_38−x_Y_x_Ni_17_ amorphous alloys.

The EDS quantitative analysis results presented in Figure 2 validate the chemical compositions of the alloys. Additionally, the elemental distribution is very homogenous for both samples and no impurities originating from the milling bowls and balls can be seen in the images.

**Figure 3:**
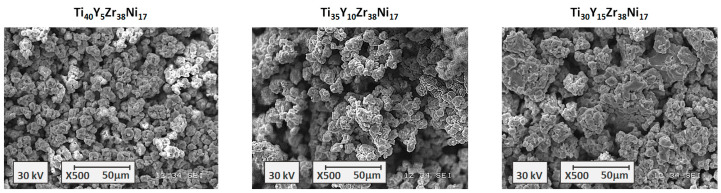
The scanning electron microscopy images for the Ti_45−z_Y_z_Zr_38_Ni_17_ amorphous alloys.

The powder microstructures of the Ti_45−z_Y_z_Zr_38_Ni_17_ alloys with z = 5, 10, and 15 are shown in Figure 3. Again, the powders consist of the agglomerates with similar diameters of 10–20 μm and the agglomerates are built with the smaller particles. No large agglomerates can be observed and all three microstructures are homogenous.

**Figure 4:**
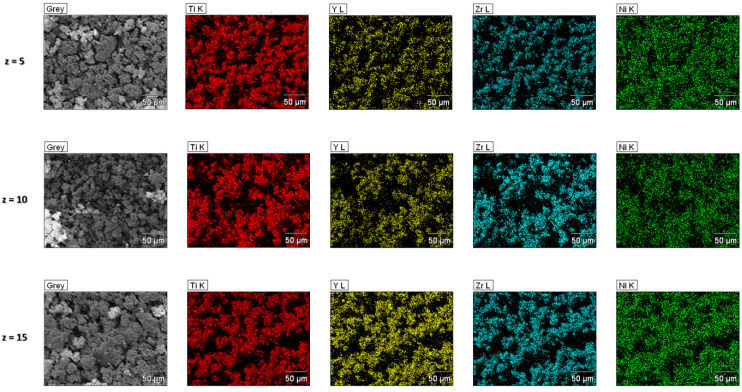
The maps of the elemental distribution for the Ti_45−z_Y_z_Zr_38_Ni_17_ amorphous alloys.

Figure 4 shows the results of the EDS analysis that confirm the chemical compositions of the Ti_45−z_Y_z_Zr_38_Ni_17_ compounds. All the elemental distributions are homogenous and impurities from the milling processes are not visible.

### 3.2. Structural Properties

**Figure 5:**
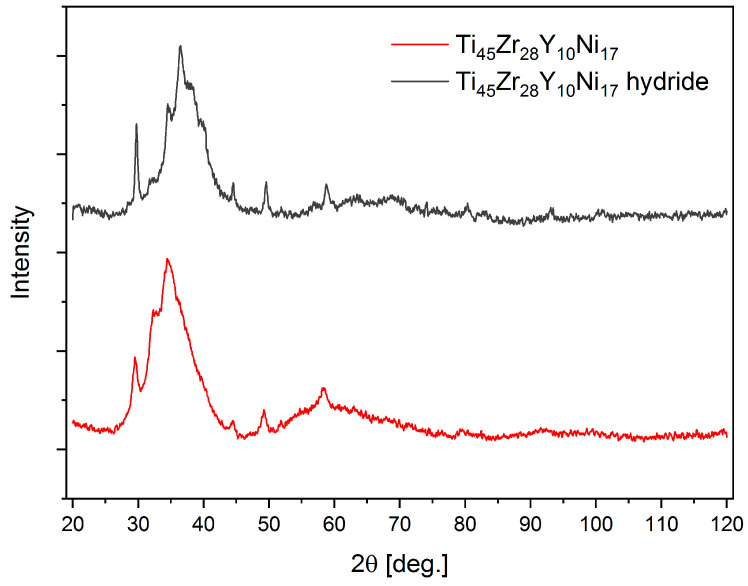
The X-ray diffraction patterns for the Ti_45_Zr_28_Y_10_Ni_17_ alloy and its hydride at the maximum hydrogen concentration.

The XRD patterns for the Ti_45_Zr_28_Y_10_Ni_17_ alloy and its hydride are shown in Figure 5. Both patterns exhibit features typical for amorphous materials, although several minor reflections can be observed. Those can be identified as corresponding to yttrium [44] and its hydride, respectively, and their presence may indicate incomplete solubility of Y in the alloy. It can be noted that the pattern for the Ti_45_Zr_28_Y_10_Ni_17_ hydride is shifted toward lower angles, which indicates an increase in the mean interatomic distances as a result of hydrogen insertion into the structure.

**Figure 6:**
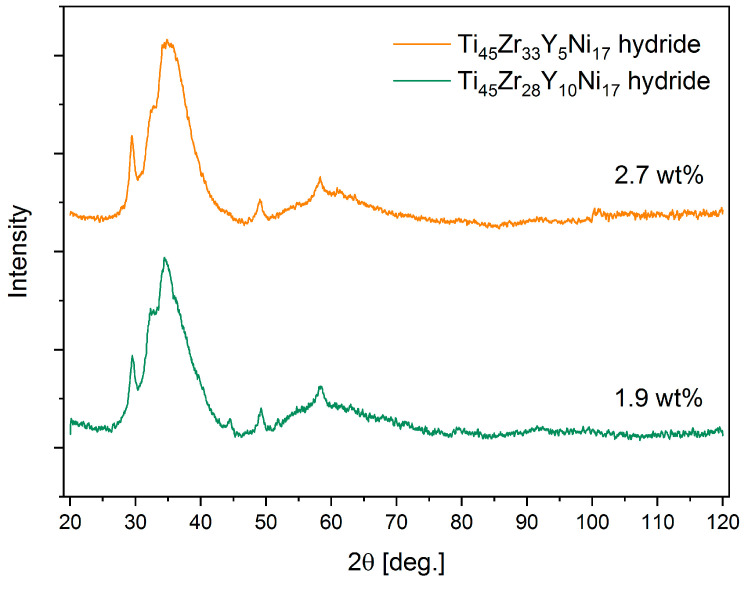
The X-ray diffraction patterns for the hydrides Ti_45_Zr_33_Y_5_Ni_17_ and the Ti_45_Zr_28_Y_10_Ni_17_ alloys at the maximum hydrogen concentrations.

The comparison of the XRD patterns for the hydrides of the Ti_45_Zr_33_Y_5_Ni_17_ and the Ti_45_Zr_28_Y_10_Ni_17_ alloy is shown in Figure 6. It is worth mentioning that the additional minor reflections are more visible for the compound containing a higher amount of yttrium.

**Figure 7:**
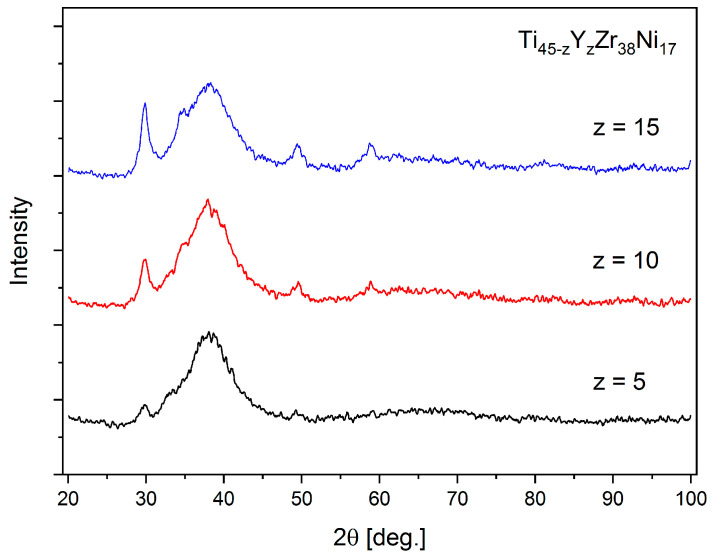
The X-ray diffraction patterns for the Ti_45−z_Y_z_Zr_38_Ni_17_ (z = 5, 10, 15) alloys.

The XRD patterns for the Ti_40_Y_5_Zr_38_Ni_17_, Ti_35_Y_10_Zr_38_Ni_17,_ and Ti_30_Y_15_Zr_38_Ni_17_ alloys are presented in Figure 7. All of them have the characteristics typical for the patterns collected for amorphous materials and, also, the minor reflections that can be attributed to yttrium not entirely dissolved in the alloys are visible. Those features become more visible with an increasing content of Y.

### 3.3. Hydrogen Sorption

**Figure 8:**
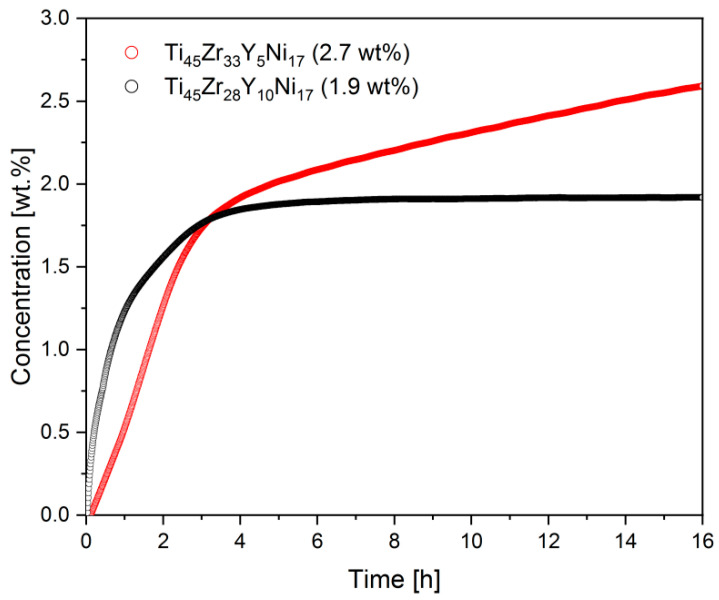
The kinetics of the hydrogenation reaction for the Ti_45_Zr_33_Y_5_Ni_17_ and Ti_45_Zr_28_Y_10_Ni_17_ alloys at 40 bar and 120 °C.

As mentioned previously, the studied alloys do not require activation, which is usually performed by cycling or annealing in a vacuum. Once exposed to hydrogen, the specimens absorbed it easily above 120 °C. Figure 8 shows the kinetics of the hydrogenation reactions for the Ti_45_Zr_33_Y_5_Ni_17_ and Ti_45_Zr_28_Y_10_Ni_17_ alloys. It can be noticed that the shapes of the curves are different and seem to be affected by the Y content in the alloy. The initial exponential growth is slowed and the curve is almost linear in the range up to 3 h for the Ti_45_Zr_33_Y_5_Ni_17_ alloy. On the other hand, the maximum hydrogen concentration for that compound was higher than for the Ti_45_Zr_28_Y_10_Ni_17_ alloy containing a higher amount of Y. It should also be noted that the kinetics were faster for the latter compound.

Additionally, it is worth mentioning that the kinetic rates are comparable to the results obtained for other recently studied novel Ti-Zr-Ni materials that were doped with vanadium [38]. However, the concentration for the Ti_45_Zr_33_Y_5_Ni_17_ is even higher than the maximum value of 2.4 wt.% for the Ti-V-Zr-Ni alloys.

Furthermore, the addition of Y to the Ti-Zr-Ni alloy allows the control of its hydrogen storage properties depending on the need. Namely, a higher concentration or faster kinetics can be achieved. Additionally, rare-earth elements such as La, Y, and Ce are known for their important role in preventing hydrogen contamination due to the surface segregation phenomenon, which leads to the formation of oxides on the surface of the alloy [45,46]. It can be assumed that the introduction of Y to the studied alloys leads to surface segregation, which, in particular, introduces the centers that facilitate cyclic hydrogenation/dehydrogenation and activation of the material. Owing to that fact better reversibility of the hydrogen absorption process can be achieved, apparently, the Y content in the Ti_45_Zr_33_Y_5_Ni_17_ alloy is optimal for the surface segregation to occur. The XRD patterns show the peaks that can be attributed to the presence of pure Y, which supports the deduction about Y precipitations on the surface of grains.

### 3.4. Deuterium Desorption

**Figure 9:**
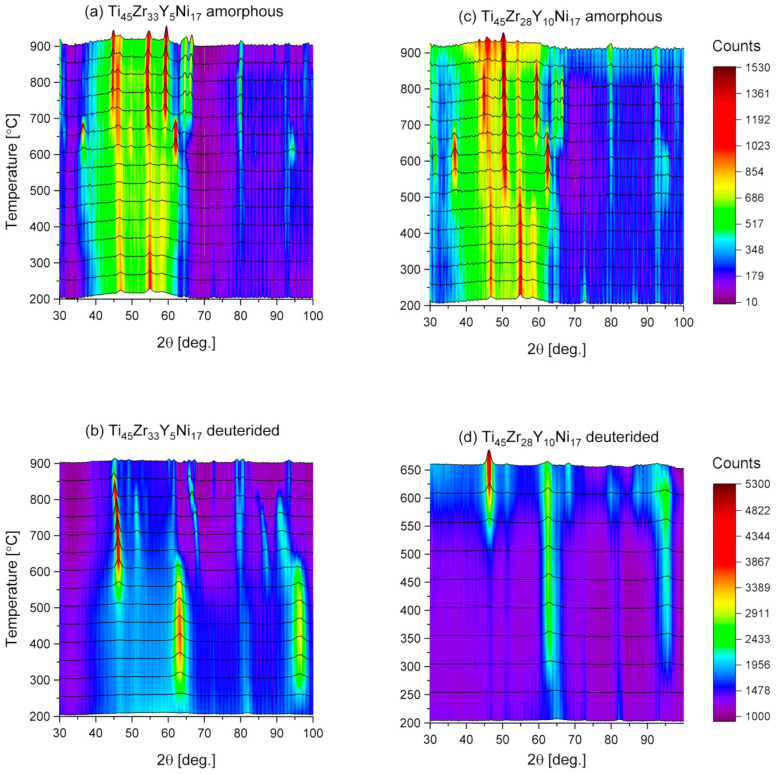
The neutron diffraction patterns for the Ti_45_Zr_33_Y_5_Ni_17_ and the Ti_45_Zr_28_Y_10_Ni_17_ amorphous alloys and the corresponding deuterides.

Figure 9 presents the neutron diffraction patterns collected during the heating of the Ti_45_Zr_33_Y_5_Ni_17_ and the Ti_45_Zr_28_Y_10_Ni_17_ amorphous alloys and the corresponding deuterides from 200 °C to 850 °C. It can be noted that the initial patterns for both alloys exhibit features typical for amorphous materials with several minor reflections observed previously in the XRD experiments. The significant changes in the patterns at around 600 °C indicate decomposition of the compounds.

The initial patterns for the deuterides are also characteristic of an amorphous structure. Two broad peaks can be observed at 63° and 96° of 2θ. The first peak represents the distribution of metal–metal distances, with a maximum value of 2.33 Å. The second peak can be attributed to the distribution of the deuterium–metal distances, with a maximum value of 1.65 Å. It was not observed in the XRD experiment due to the fact that, although deuterium is a good neutron scatterer, low electronic charge around deuterium and hydrogen atoms makes the observation with the use of X-rays not viable. The presence of those two peaks is direct evidence that deuterium is not associated with any particular component of the amorphous matrix and is an equal constituent of the alloy. What is more, such two peaks indicate the occurrence of the novel glassy quasicrystalline phase that was also detected for the deuterides of the Ti_45_Zr_38_Ni_17−x_Co_x_ (x < 8) [39] and the Ti_45−x_V_x_Zr_38_Ni_17_ (5 ≤ x ≤ 40) amorphous alloys [38].

It can be seen in Figure 9 that deuterium desorption started at around 600 °C. The changes in the patterns indicate that the compounds decomposed into simple phases and then transformed into the c-phase (cubic). Such behavior means that the desorption of deuterium is associated with the crystallization of the amorphous alloy and was previously observed for the similar Ti-V-Zr-Ni and Ti-Zr-Ni-Fe materials [38,39].

**Figure 10:**
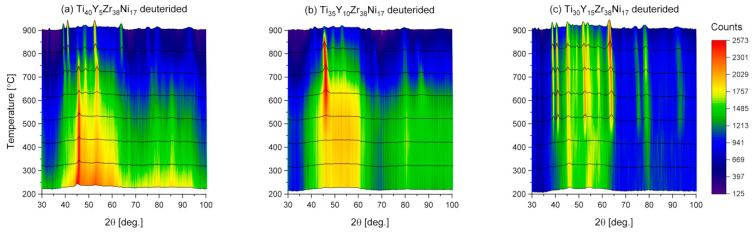
The neutron diffraction patterns collected during heating of the Ti_40_Y_5_Zr_38_Ni_17_, Ti_35_Y_10_Zr_38_Ni_17_, and Ti_30_Y_15_Zr_38_Ni_17_ deuterides.

The ND patterns collected during heating of the Ti_40_Y_5_Zr_38_Ni_17_, Ti_35_Y_10_Zr_38_Ni_17_, and Ti_30_Y_15_Zr_38_Ni_17_ deuterides from 200 °C to 900 °C are presented in Figure 10. The characteristics of the initial patterns are similar to those shown in Figure 9; however, the peaks that can be attributed to the yttrium deuteride (46° and 80° of 2θ) are more visible. Again, two broad peaks can be observed at 54° and 95° of 2θ. The first peak relates to the distribution of the metal–metal distances with the maximal value of 2.65 Å, which is higher than the value determined for the Ti_45_Zr_38−x_Y_x_Ni_17_ series. That can be explained by the fact that Y atoms with higher diameters of 180 m are substituted for Ti atoms with diameters of 140 pm. Additionally, that observation confirms that Y is present in the alloys not only as precipitation but as their constituent. The second peak can be attributed to the distribution of the deuterium–metal distances with the maximal value of 1.66 Å. Similarly to the Ti_45_Zr_38−x_Y_x_Ni_17_ series, the occurrence of those two peaks yields proof that deuterium is an equal constituent of the Ti_45−z_Y_z_Zr_38_Ni_17_ deuterides and the glassy quasicrystalline phase is observed for those compounds.

Deuterium desorption for the Ti_45−z_Y_z_Zr_38_Ni_17_ deuterides started at around 500 °C. The changes in the patterns indicating that the compounds decomposed into simple phases and transformed into the c-phase are clearly visible especially in Figure 10c for the compound with the highest Y content at higher temperatures.

### 3.5. In-Situ Neutron Diffraction under High Deuterium Pressure

**Figure 11:**
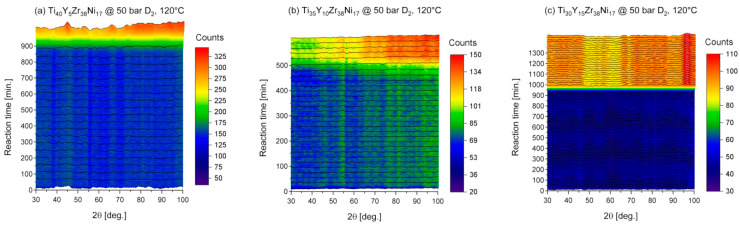
The in-situ neutron diffraction patterns for the Ti_40_Y_5_Zr_38_Ni_17_, Ti_35_Y_10_Zr_38_Ni_17_, and Ti_30_Y_15_Zr_38_Ni_17_ amorphous alloys under deuterium pressure of 50 bar.

The in-situ neutron diffraction experiments under high deuterium pressure were performed to gain a deeper understanding of the mechanism behind the synthesis of the amorphous deuterides. The reactions were conducted for the Ti_40_Y_5_Zr_38_Ni_17_, the Ti_35_Y_10_Zr_38_Ni_17_, and the Ti_30_Y_15_Zr_38_Ni_17_ amorphous samples at an initial pressure of 50 bar and a relatively low temperature of 120 °C in order to extend the duration of the experiments, which allowed the collection of the data with proper statistics. The results of the experiments are presented in Figure 11. The initial patterns are consistent with the results of the XRD measurements with the features typical for the amorphous alloys and several minor reflections that can be attributed to the presence of not entirely dissolved Y. It can be seen in Figure 11 that the reaction rates were low at the beginning and then rapid deuteriding occurred. The reaction was fastest for the Ti_35_Y_10_Zr_38_Ni_17_ alloy (deuteriding after 500 min.) and the slowest for the Ti_30_Y_15_Zr_38_Ni_17_ alloy (deuteriding after 1000 min.). It can also be noticed that the reaction for the sample with the lowest content of Y (the Ti_40_Y_5_Zr_38_Ni_17_) was slower than for the Ti_35_Y_10_Zr_38_Ni_17_ specimen. The latter is in accordance with the kinetics of hydrogenation of the Ti_45_Zr_33_Y_5_Ni_17_ and Ti_45_Zr_28_Y_10_Ni_17_ amorphous alloys, for which the slower reaction rate was observed also for the sample with the lower content of Y.

Deuteriding of the alloys resulted in the occurrence of two broad peaks at 54° and 95° of 2θ, which is consistent with the results of the deuterium desorption experiments performed for those alloys and discussed in Section 3.2. The first peak associated with the distribution of the metal–metal distances is shifted toward lower angles at the later stages of the reactions, which can be explained by an increase in those distances with deuterium entering the structure. The analogous effect was observed previously for the Ti_45−x_V_x_Zr_38_Ni_17_ (5 ≤ x ≤ 40) amorphous alloys [38].

In addition, an increase in the background can be noticed after the start of deuteriding. That phenomenon is related to the presence of hydrogen, which cannot be avoided. The typical deuterium purity is equal to 98% and hydrogen accounts for 1.99% of the remaining impurities (2%). Unfortunately, even such a small amount of hydrogen that is absorbed by the sample leads to the occurrence of the background signal because of the high incoherent scattering cross section of H_2_.

## 4. Conclusions

The results of the XRD and ND structural studies reveal that the Ti_45_Zr_38−x_Y_x_Ni_17_ (x = 5, 10) and the Ti_45−z_Y_z_Zr_38_Ni_17_ (z = 5, 10, 15) alloys and their hydrides/deuterides are mostly amorphous with a small addition of not entirely dissolved yttrium.

It was shown that the highest hydrogen concentration can be reached for the Ti_45_Zr_33_Y_5_Ni_17_ alloy and is equal to 2.7 wt.%. The hydrogen concentration varies with the x and z values and, consequently, it depends on the amount of Y in the alloy and whether Y is substituted for Zr or Ti. Therefore, an important conclusion can be drawn that the hydrogen storage properties of the parent Ti_45_Zr_38_Ni_17_ alloy can be precisely altered by doping with yttrium.

In addition, it was established that the kinetics of the hydrogenation/deuteriding reaction also depends on the x and z values. The highest reaction rate can be achieved for the alloys with moderate Y doping (x = 10 and z = 10) for both the Ti_45_Zr_38−x_Y_x_Ni_17_ and Ti_45−z_Y_z_Zr_38_Ni_17_ alloys.

What is more, it was shown that the mean interatomic distances increase as a result of hydrogenation/deuteriding. The results of the desorption and in-situ experiments confirm that hydrogen/deuterium is an equal constituent of the amorphous alloy not associated with any specified element of the alloy matrix, which is another important achievement of this work. In addition, it is demonstrated that the hydrides/deuterides decompose into simple phases and transform into the c-phase as a result of desorption at temperatures higher than 600 °C.

Finally, the presence of the novel glassy quasicrystalline phase is confirmed for the deuterides of the Ti_45_Zr_38−x_Y_x_Ni_17_ and Ti_45−z_Y_z_Zr_38_Ni_17_ alloys. The results of the studies presented in this paper are an important step in the search for novel hydrogen storage materials and the understanding of their interaction with hydrogen. In the long run, such research brings us closer to development of clean energy storage systems based on hydrogen as an energy carrier.

## Data Availability

The original contributions presented in the study are included in the article, further inquiries can be directed to the corresponding author.
